# Supplementary material for: Differential activity and expression of human 5β-reductase (AKR1D1) splice variants
Source: J Mol Endocrinol. 2021 Jan 12;66(3):181–94. doi: 10.1530/JME-20-0160 (PMC7965358; doi:10.1530/JME-20-0160)
Supplement: Suppl. Figure 1: Schematic demonstrating the formula used to determine relative mRNA values of AKR1D1 splice variants using qPCR Taqman probes spanning three different exon-exon junctions. [file supplementary_figure_1.pdf]

Suppl. figure 1

a

$$\text{Relative } \mathit{AKR1D1\text{-}001} = \frac{\text{Total } \mathit{AKR1D1} - (\mathit{AKR1D1\text{-}002} + \mathit{AKR1D1\text{-}006})}{\text{Total } \mathit{AKR1D1}} \times 100$$

$$= \frac{\text{Probe}_{\text{EXONS 3-4}} - \text{Probe}_{\text{EXONS 7-8}}}{\text{Probe}_{\text{EXONS 3-4}}} \times 100$$

$$\text{Relative } \mathit{AKR1D1\text{-}006} = \frac{\text{Total } \mathit{AKR1D1} - (\mathit{AKR1D1\text{-}002} + \mathit{AKR1D1\text{-}001})}{\text{Total } \mathit{AKR1D1}} \times 100$$

$$= \frac{\text{Probe}_{\text{EXONS 3-4}} - \text{Probe}_{\text{EXONS 5-6}}}{\text{Probe}_{\text{EXONS 3-4}}} \times 100$$
