# Supplementary figures and images for: Differential activity and expression of human 5β-reductase (AKR1D1) splice variants
Source: J Mol Endocrinol. 2021 Jan 12;66(3):181–94. doi: 10.1530/JME-20-0160 (PMC7965358; doi:10.1530/JME-20-0160)

# Suppl. figure 2

a

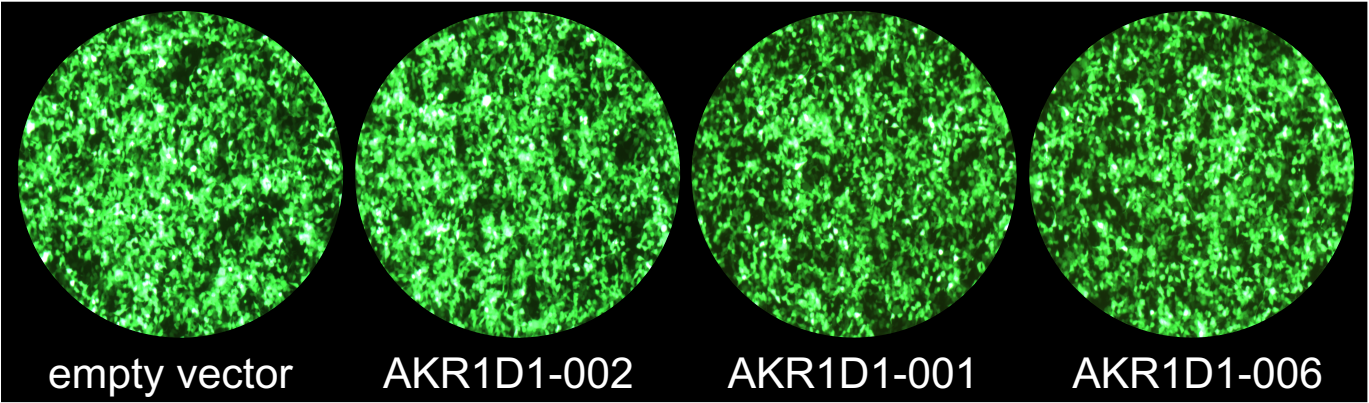

b

Green Fluorescence Protein

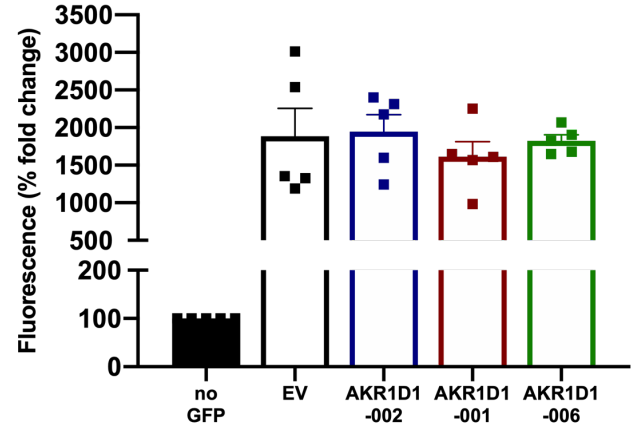

c

*Hs00973526\_g1*

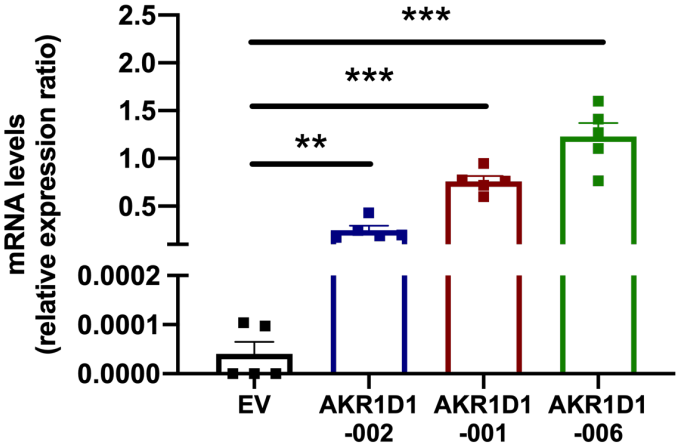

d

*Hs00973528\_gH*

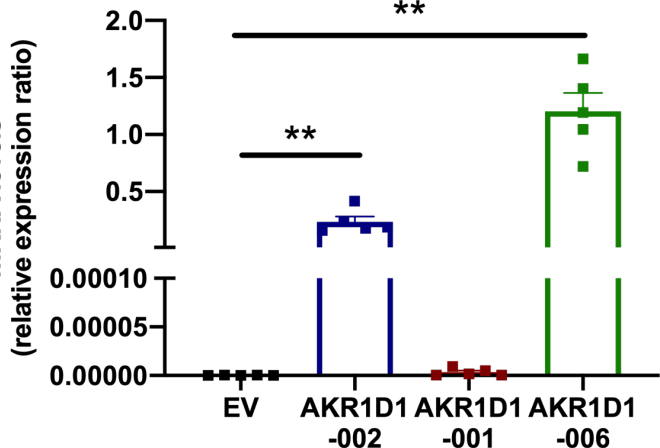

e

*Hs00975611\_m1*

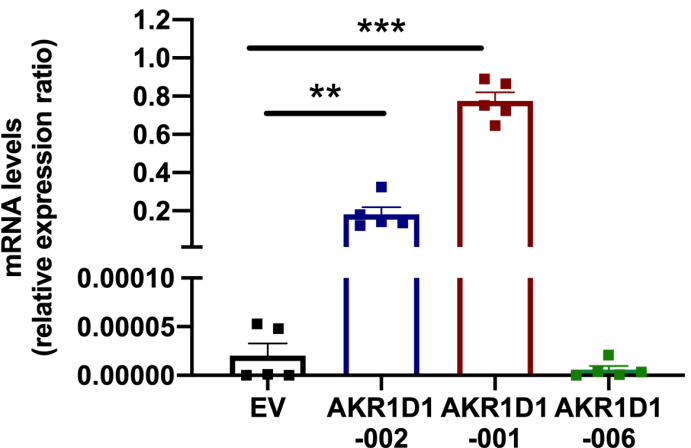

Supplement: Suppl. Figure 2: (a) Representative microscopy photos demonstrating Green Fluorescence Protein (GFP) signal following co-transfection of GFP and AKR1D1-SV constructs. (b) Relative GFP fluorescence levels following co-transfection of GFP and AKR1D1-SV constructs (n=5). (c-e) mRNA over-expression leve [file supplementary_figure_2.pdf]
